# Dysentery*Read before the Medical Union.

**Published:** 1884-02

**Authors:** E. T. Dorland


### BUFFALO

### Medical and Surgical Journal.

Vol. XXIII. FEBRUARY, 1884. No. 7.

### Original Communications.

### Dysentery.*

By E. T. Dorland, M. D.

It is a curious and interesting fact, confirmed by the concurrent testimony of medical observers from the age of Hippocrates to the present day, that the different seasons of the year are marked by an aptitude to certain determinate diseases in the human system. Nor is this any marvel, when we consider the numerous, great and sudden meteorological changes of the successive seasons, together with the corresponding diversities in the vast vegetable kingdom, with which we hold such intimate connection and relation. The conclusion is pressed upon us, irresistably, that the sensitive and wonderfully complicated organization of man cannot possibly exist in the midst of these ever varying circumstances uninfluenced and unaffected, but that for weal or woe it must be powerfully under their control.

Owing undoubtedly to the nature and degree of their changes, the same seasons in different years in similar latitudes vary widely from one another in the form, type, severity and mortality of their maladies. The same is true of the age, and occasion-

*Read before the Medical Union.

ally the sex of those whom they select for their victims. The severity varies so much, that while some are marked by nothing inordinate in these respects, exciting no disease but such as is readily amenable to medical treatment, or so mild, even as to pass by almost unnoticed, others are marked by the most appalling traits, generating complaints that bid defiance to the best directed skill of the most enlightened physicians.

Providentially removed from the sweltering heat and resulting pestiferous influences of the southern climes, and proportionately free from large cities, those great national curses, as they have been designated, which congregate poverty and vice, too often converting them into vast pest houses; it has been the good fortune of this part of the Empire State to enjoy a comparative immunity from those seriously malignant epidemic diseases. It does happen, however, at distant periods of time, that the otherwise steady current of health has been rudely interrupted; and, as if to take vengeance for past exemption, sickness and death have run riot in our midst.

Such was the case in the epidemic of dysentery that visited this city and the adjacent country in the autumn of 1853. I was located at that time at Buffalo Plains, as resident physician of the almshouse, and attended a large number of cases in that vicinity, as well as caring for all occurring within the institution itself. Being greatly interested in the cases as young practitioners are wont to be, and taking notes of every case at the time, I have felt that it might not be altogether unprofitable to present to this society a brief synopsis of my views of the diagnosis and treatment of dysentery.

It is true, the epidemic referred to occurred thirty years ago, yet I can state that the few typical cases that have come under my observation since that time have not changed the views then held of the true nature and proper treatment of this disease. Great changes and decided improvements in our materia medica have taken place since that day, but the indications of treatment remain the same, virtually unchanged by the lapse of time. I

will here state that I believe it to be conceded by most observers, that malignant epidemics of dysentery have commonly been preceded by an unusual prevalence of choleric maladies. In this instance, as you may remember, cholera raged here severely in 1852, and not a little during the summer of 1853, and it was on the subsidence of the cholera that the epidemic referred to made its appearance. I shall not, in this brief paper, attempt to treat upon the pathology of dysentery, assuming that you are all better informed upon that subject than myself, venturing to state, in passing, that I believe pathologists mainly agree that the affection consists primarily of inflammation of the solitary and sometimes of the other glands and follicles belonging to the intestinal mucous membrane. I propose to treat briefly of the symptoms of the mild and severer forms of dysentery, and the treatment appropriate to each. The milder I shall denominate dysenteric diarrhœa, and the severer, dysentery. The symptoms characterizing a case of dysenteric diarrhœa are, of course, modified somewhat by circumstances, as age, temperament, habits, etc. But as a rule, the following would be an approximate description, having reference especially to the epidemic of 1853, though applicable, according to my observation, to the disease in general, whenever and wherever found.

The first deviation from health, noticed by the patient, would be a moderate and not very distressing diarrhœa. Fever was absent in the majority of instances, and the pulse, skin, urinary secretion, tongue and appetite were very often nearly or quite normal, the only indication of derangement in the system being the frequentand loose stools, together with some pain or uneasiness in the bowels, felt principally just previous to an evacuation. The stools would be sometimes wholly fecal, and again partly fecal and partly mucous and blood. But usually within from three to five days either convalescence was established, or the case had developed into one of unmixed dysentery. I invite your attention especially to the marked difference in the condition of the system in the early stage of the two forms of the disease.

The symptoms characterizing a case of primary unmixed dysentery, experience would give as follows: A case of primary dysentery is often ushered in by a chill, followed in a short time by a febrile reaction of considerable severity. To these soon succeed a feeling of weight and distress in some portion of the abdomen, centering sooner or later about the pubic and left inguinal regions, while over these regions there is more or less tenderness on pressure. The febrile action, whether preceded by chill or not, is usually high and continuous with evening exacerbations; the appetite capricious, and ofttimes complete anorexia exists. Nausea is a common attendant, though vomiting, unless from over-burdened stomach, is rare. The tongue is generally, but not in all cases, coated. The coating is sometimes a light, thin, narrow strip only in the center, or a thin extended lining, of a white color; at other times the lining is thick and of a brownish cast, but very frequently there is little, if anything, abnormal in the state of the tongue. Some writer has remarked sagely, I think, that the state of the tongue is a poor index to the degree or extent of inflammation in the large intestines alone, although a good one generally in diseases of the small intestines and stomach. I am sure, in my own experience, that I have rarely seen the red, dry, parched appearance of that organ in genuine dysentery, though commonly so in inflammations of the intestinal tube higher up.

The alvine evacuations are numerous, thin and scanty; they are very variable in their color and composition, sometimes consisting chiefly of blood, then of mucous, then of blood and mucous intermixed, then of a yeast-like substance, and then again of a peculiar greenish matter, resembling lumps of flesh stained with bright green pigment. The last appearance is produced, according to some authors, by a mixture of coagulated blood and bile. Tormina and tenesmus are distressing symptoms. The pulse ordinarily continues accelerated, full and tense all through the earlier stages, unless the case takes on a sinking typhoid character. Other symptoms characteristic of inflammatory affections in general I need not here enumerate, but pass on to the consideration of what I had more prominently in view when I began this paper, viz., the treatment. There were two opposing theories of treatment in vogue at the time of the epidemic referred to, and each was earnestly defended by leading practitioners of the day; one was the opium plan, and the other the purgative.

The purgative theory was taught and practiced by Prof. Austin Flint, and his success in treating this particular disease was considered by all, whether professional or non-professional, as anything but flattering. The same ill-success attended his treatment of cholera, which, as before stated, was very severe with us in 1852 and 1853. Dr. Flint held that both cholera and dysentery were primarily and principally due to an obstruction of the portal circulation. That the wicked liver was the chief cause of all the trouble; and holding that opinion, the sheet-anchor of treatment, with him, was calomel first, last and all the time, and in addition to the calomel, every two or three days a saline cathartic, or rhubarb or castor oil would be given. The use of opium, to any considerable extent, was deprecated on the theory that it tended to check the action of the liver and other secretory organs, and was prescribed only when pain was continuous and severe, or tenesmus extremely distressing, as an unavoidable necessity. And I find similar arguments used against the use of opium in dysentery by some modern writers, as, for instance, you will find in the "Dictionary of Medicine," by Quain (a recent work), a very full and able article on dysentery, its pathology and treatment, wherein the following statement appears in reference to the use of opium: "Opium, by the mouth, is seldom required. When swallowed it locks up the secretions of the liver, pancreas and alimentary mucous membrane, rather favoring than reducing the inflammation of the solitary and tubular glands. These bad effects counterbalance the benefits derived from the sleep, diminution of peristaltic action, and temporary decrease of tormina, etc."

Prof. Flint was not the only eminent practitioner who taught the purgative theory at that day. Professors Pitcher and Denton, of the Ann Arbor school, both taught it at the course of lectures which I attended there during the winter of 1853 and 1854. At that time the great body of American physicians were divided upon it, and it formed a sharp line of demarcation between the English and French schools of practice, the latter contending for, the former against it. Who shall decide where so many doctors disagree? It is not for me to assume the station of the umpire. I speak only for myself, and I would not, therefore, without reason, foist my notions of the subject on the consideration of the society. In the discussion of questions of so great importance as those involving the saving of human lives, we have a right to be outspoken in the expression of our opinions. There were, at that time, in Buffalo, many beside Dr. Flint who believed in the purgative theory. Dr. Pratt, my preceptor, told me that Dr. Barnes practiced that system for a while, and lost nearly all his cases. Their theory was that catharsis relieved the hyperæmic condition of the capillary vessels, and promoted the general secretions of the intestines, and as a consequence inflammatory action would be abated. It remains for me to describe the plan of treatment adopted by many of us, and which was certainly very successful, and which I believe to be the proper treatment of dysentery, in my judgement. It is of vital importance, when called to a case of supposed dysentery, to ascertain whether it be of the diarrhœal form, indicated by more or less fecal matter in the stools, an absence of any great constitutional disturbance, a preceding diarrhœa, etc., in which case very little treatment is usually required. Rest, a proper attention to the diet, which should consist mainly of milk, milk porridge, rice, mutton broth and the like, a free use of alkalies, some simple diarrhœa mixture with sufficient anodynes to relieve pain and control the peristaltic action of the bowels, makes up, as a rule, the sum total of treatment necessary for this form of the disease. But when called to a case of real typical dysentery, as indicated by stools containing little or no fecal matter, high febrile excitement, much tenderness on pressure over the bowels, etc., as before described, our course of treatment was as follows: First a full dose of calomel, followed in a few hours by a dose of castor oil to empty the intestinal canal of all undigested food and fecal matter, immediately after which we put the patient upon full doses of opium, and from that time on it was opium first, last and all the time, the advocates of the opium plan of treatment believing it as absurd to claim that cathartics promote the secretion of the intestinal glands in the inflamed condition of dysentery, as that irritants applied to inflamed mammæ promote the secretion of milk. On the contrary, we maintain that opium, beside relieving pain, procuring rest and controlling the peristaltic action of the bowels, really promotes secretion by diminishing the morbid sensibility of the bowels to the matters they contain, and thereby greatly relieving the spasmodic constriction of the vessels involved in the inflammatory action. But to return; the opiates, as thesheet-anchor of treatment, we continued as long as there was any considerable inflammatory action remaining, and almost invariably with the most happy results. Auxiliary measures, such as hot fomentations and turpentine stupes to the bowels, alkaline drinks, opium suppositories to relieve tenesmus, etc., were, of course, made use of whenever rationally indicated.

I claim it to be as logical to keep the system under the influence of opium in acute dysentery, as we all, I believe, concede it to be in enteritis.

From my own experience, and from the knowledge derived by observation of the experience of others, I fully believe the plan so highly lauded by some physicians, of giving frequently, in dysentery, cathartic doses of calomel, castor oil, magnesia, rhubarb, rochelle salts, etc., to be uncalled for, injurious and cruel, and therefore decidedly bad practice.

The rationale I present to you in conclusion, and as a "summing up of the case."

The purgative treatment is irrational; first, because it is diametrically opposite to that cardinal law of both medicine and surgery, derived from the example of nature herself, that injured and inflamed parts be kept in the most passive and quiescent state, and that the exercise of their functions be diminished as much as possible. Witness the complete rest enjoined in sprain of the ankle, or inflammation of the knee-joint, the prohibition of the use of the eye in opthalmia, the suppression of noise and the exclusion of light in phrenitis, the forbidance of any article making requisition on the digestive powers of the stomach in gastritis, etc.

Secondly, because it is directly opposed to another law in medicine and surgery, which forbids the application of irritants to an already irritated surface. To this rule there exist only rare exceptions, which do not apply to the question under consideration.

Thirdly, because the administration of purgatives is very apt to have the effect, especially in persons of nervous temperament and weak digestive organs, to unsettle and nauseate the stomach and render it incapable of the retention of food. In compelling the patient to unnecessary stools, it must also contribute to harmful exhaustion and suffering.

And fourthly and lastly, because experience has pretty satisfactorily proved that those hardened fæces termed scybalæ, which were formerly supposed to exist in the bowels in nearly all cases of dysentery, and to dislodge which purgatives were originally employed, are found only in a few cases, and, when existing, their unaided and unprovoked passage is less disturbing and harmful than the action of cathartics.

